# Diabetes mellitus, thyroid dysfunctions and osteoporosis: is there an association?

**DOI:** 10.1186/2251-6581-12-38

**Published:** 2013-07-08

**Authors:** Mostafa Qorbani, Hamid Reza Bazrafshan, Mehrdad Aghaei, Hossien Shadpour Dashti, Aziz Rezapour, Hamid Asayesh, Rasool Mohammadi, Younes Mohammadi, Hossein Ansari, Morteza Mansourian

**Affiliations:** 1 Department of Public Health, Alborz University of Medical Sciences, Karaj, Iran; 2 Non-Communicable Diseases Research Center, Endocrinology and Metabolism Research Institute, Tehran University of Medical Sciences, Tehran, Iran; 3 Bone joints & Connective Tissue Research Center, Golestan University of Medical Sciences, Gorgan, Iran; 4 Hospital Management Research Center, Iran University of Medical Sciences, Tehran, Iran; 5 Department of Medical Emergency, Qom University of Medical Sciences, Qom, Iran; 6 Department of Epidemiology, School of Public Health, Shahid Beheshti University of Medical Sciences, Tehran, Iran; 7 Department of Epidemiology & Biostatistics, School of Public Health, Hamadan University of Medical Sciences, Hamadan, Iran; 8 Health Promotion Research Center, Zahedan University of Medical Sciences, Zahedan, Iran; 9 Department of Health Education, Ilam University of Medical Sciences, Ilam, Iran

**Keywords:** Osteoporosis, Bone density, Diabetes mellitus, Thyroid diseases

## Abstract

**Background:**

Osteoporosis is the most common metabolic bone disease with complicated, multifactorial and heterogenic nature that has no known pathological cause. As the role of Diabetes Mellitus (DM) and thyroid dysfunctions in the prevalence of osteoporosis is not exactly known, therefore this study was designed to evaluate the probable association between osteoporosis with DM and thyroid dysfunctions in Iranian patients.

**Methods:**

In this cross-sectional study, 300 subjects out of the total number of patients referring to Gorgan bone densitometry centers (3000subjects) were selected via random sampling method in 2009. Individual characteristics, DM, thyroid dysfunctions and densitometry results were collected from densitometry records. Data analysis was carried out by SPSS version 16 software and by using Chi square and T-test. The level of significance in all tests was considered 0.05.

**Results:**

The mean of T-score in lumbar and femoral areas of diabetic patients were -0.87 ± 1.08 and -1.94 ± 1.33 and in patient with thyroid dysfunctions was -0.80 ± 1.09 and -1.64 ± 1.24 respectively. The mean of BMD in lumbar and femoral areas of diabetic patients were 0.96 ± 0.19 and 0.75 ± 0.19 and in patient with thyroid dysfunctions were 0.96 ± 0.17 and 0.76 ±0.19 respectively. The mean of BMI in osteoporotic subjects in the lumbar and femoral areas were 25.94 ± 5.62 and 26.95 ± 5.20 respectively. The association between BMI and BMD in the lumbar and femoral areas were statistically significant, but the association between DM and thyroid dysfunctions with BMD and T-score in the femoral and lumbar areas was not statistically significant (P-value > 0.05).

**Conclusion:**

The results of current study show that there is no association between DM and thyroid dysfunctions with osteoporosis.

## Introduction

Osteoporosis is the most common metabolic bone disorder that is characterized by reduction of bone mass without alteration in the composition of bone, leading to fractures. The most obvious subclinical symptom of this disorder is the fracture in the vertebral and femoral neck bone [1]. Osteoporosis cause approximately 2 million fractures annually, which decrease quality of life, increased disability and even death [2]. The National Osteoporosis Foundation estimated that approximately 45 million persons over the age of 50, in 2002, in the United States were at risk for fracture due to osteoporosis and they estimated that in 2020, that number is over 60 million [3]. The osteoporosis has a complex and heterogenic nature the cause of which is not yet known. The known risk factors for osteoporosis are menopause, age, family history of fracture, race, immobility, smoking, corticosteroids, and deficiency of some nutrients such as calcium and vitamin D [4, 5]. The effect of some extrinsic causes of osteoporosis, including genetic disorders, autoimmune diseases (e.g. lupus, rheumatoid arthritis and etc.) endocrine disorders (e.g. Diabetes Mellitus (DM), Cushing’s syndrome, hypothyroidism and hyperparathyroidism) gastrointestinal disorders (e.g. celiac disease, gastric bypass, GI surgery, inflammatory bowel disease, pancreatic disease and cirrhosis), and hematologic disorders (e.g. hemophilia, sickle cell disease and thalassemia) are not well known [6].

DM is accompanied by a comprehensive list of chronic complications that affect almost all tissues. Musculoskeletal complications in DM have been identified since the beginning of the 20^th^ century [7]. As DM and osteoporosis are prevalent and have especial socio-economic importance in society, the evaluation of the interaction of these two diseases has been recognized even more. The important question is that whether DM is a risk factor for osteopenia and osteoporosis, or these disorders are complication of DM. However, the results of bone mineral density (BMD) in type II DM are different and sometimes contrary [8–11]. In some studies no difference has been observed in BMD between patients with type II DM and healthy individuals. A number of studies have reported lower BMD in type II diabetic patients than normal population, while there are still others in which the BMD has been higher in diabetics in comparison with normal population [6].

The most common causes of secondary osteoporosis can be mentioned as follows: increase endogenic and exogenous glucocorticoids, hyperthyroidism, hyperparathyroidism, and vitamin D deficiency [12–19]. Hyperthyroidism causes bone erosion which has been reported to be an important reason for the increase in the brittleness of collagenase tissue, as well as the decrease in BMD. The role of thyroid hormones in bone metabolism is not identified, but the impact of T3 on osteoclasts has been suggested in bone erosion [20]. As the role of DM and thyroid dysfunctions in the prevalence of osteoporosis is not exactly known, therefore this study was designed to evaluate the probable association between osteoporosis with DM and thyroid dysfunctions among subjects referring to Gorgan bone densitometry centers, north-east of Iran.

## Methods

Among 3000 subjects referring to Gorgan densitometry centers (two centers) in 2009, 300 subjects were selected via random sampling method. The study was approved by ethic committee of Golestan University of Medical Sciences. BMD of the lumbar spine (L2 and L4) and proximal femur (femoral neck) was measured by Dual Energy X-Ray Absorptiometry (DXA) (Norland XR-46). According to the WHO criteria by using T-Score of lumbar and femoral BMD, osteoporosis (T-score less than -2.5 of standard deviation in DXA densitometry) and osteopenia (T-Score between -1 to -2.5 of standard deviation in densitometry) were defined [21]. The demographic data including age, weight, height, and medical history such as endocrine disorders (DM and thyroid dysfunctions) was collected via interview with specialist. Weight was recorded in light clothing to the nearest 0.1 kg on a SECA digital weighing scale (SECA, Germany) and height was measured without shoes to the nearest 0.1 cm. Body mass index (BMI) was calculated from weight and height [BMI = weight (kg) /height (m^2^)]. Four categories were defined for BMI: underweight (BMI < 18.5 kg/m^2^), normal weight (BMI = 18.5–25 kg/m^2^), overweight (BMI = 25–30 kg/m^2^), and obese (BMI > 30 kg/m^2^). In the analysis BMI was considered as a continuous and categorical variable. Thyroid disorders and DM were considered as binary variables and BMD were regarded as continuous and categorical variable (normal, Osteopenia, and osteoporosis).

Data were analyzed using independent T-test, ANOVA and Tukey Post hoc test, Chi square test and univariate and multiple linear regression. Analysis was done with SPSS version 16 software. A P-value less than 0.05 were considered as statistically significant.

## Results

Among 300 participants studied, 260 cases (86.6%) were women, 38 cases (12.7%) were diabetic, and 28 cases (9.3%) suffered from thyroid dysfunctions. The mean age and BMI of the participants was 52.7 ± 14.42 years and 28.14 ± 5.42 kg/m^2^ respectively. The mean BMD in the lumbar and femoral areas was 0.92 ± 0.19 g/cm² and 0.77 ± 0.16 g/cm² respectively. The mean BMI in osteoporotic, osteopenic, and normal subjects in the lumbar area was 25.94 ± 5.62 kg/m^2^, 27.71 ± 4.93 kg/m^2^ and 29.38 ± 5.49 kg/m^2^ respectively which was statistically significant (P-value < 0.01). Post hoc test showed that this difference was statistically significant only between normal and osteoporotic cases (P- value <0.01). The association between BMI categories and BMD in lumbar and femoral areas was statistically significant (P-value: 0.01) (Table 1).

**Table 1:** **The association between BMI categories and BMD status in study population in lumbar and femoral areas**

		BMI categories^1^				P-vale
Area	Status	Underweight	Normal	Overweight	Obesity	
Lumbar	Normal	2 (0.7)	20 (6.7)	5 (16.7)	57 (19)	0.01
	Osteopenia	5 (1.7)	35 (11.7)	47 (15.7)	34 (11.3)	
	Osteoporosis	5 (1.7)	18 (6)	15 (5)	12 (4)	
Femoral	Normal	1 (0.3)	12 (4)	34 (11.3)	37 (11.3)	0.01
	Osteopenia	6 (2)	25 (8.3)	44 (14.7)	40 (13.3)	
	Osteoporosis	5 (1.7)	36 (12)	34 (11.3)	26 (8.7)	

^1^BMI categories: underweight: BMI < 18.5 kg/m^2^, normal weight: BMI; 18.5–25 kg/m^2^, overweight: BMI; 25–30 kg/m^2^, obese: BMI > 30 kg/m^2^.

Data are presented as number (%).

Mean of T-score and BMD in participants according to DM and thyroid dysfunctions is presented in Table 2. Mean of T-score in lumbar and femoral areas of diabetic patients was -0.87 and -1.94 and in patient with thyroid dysfunctions was -0.80 and -1.64 respectively. Mean of BMD in lumbar and femoral areas of diabetic patients was 0.96 and 0.75 and in patient with thyroid dysfunctions was 0.96 and 0.76 respectively. The association between DM and thyroid dysfunctions with BMD and T-score in the femoral and lumbar areas was not statistically significant (P-value > 0.05).

**Table 2:** **Mean of T-score and BMD in participants according to DM and thyroid dysfunctions in lumbar and femoral areas**

Area	Status	Diabetes		P-value	Thyroid dysfunctions		P-value
		Yes	No		Yes	No	
Lumbar	T-score	-0.87 (1.08)	-1.10 (1.21)	0.27	-0.80 (1.09)	-1.09 (1.20)	0.24
	BMD	0.96 (0.19)	0.92 (0.20)	0.26	0.96 (0.17)	0.92 (0.20)	0.29
Femoral	T-score	-1.94 (1.33)	-1.72 (1.32)	0.33	-1.64 (1.24)	-1.75 (1.31)	0.68
	BMD	0.75 (0.19)	0.78 (0.16)	0.38	0.76 (0.19)	0.78 (0.16)	0.60

Data are presented as mean (SD).

Among 38 diabetic subjects, 17 subjects (5.7%) were osteopenic, and 5 subjects (1.7%) were osteoporotic in the lumbar area. In the femoral area, 19 subjects (6.3%) were osteopenic and 14 subjects (4.7%) were osteoporotic which was not statistically significant in both areas (P > 0.05) (Table 3).

**Table 3:** **The association of DM, thyroid dysfunctions and BMD status in study participants in lumbar and femoral areas**

Area	Status	Diabetes		P-value	Thyroid dysfunctions		P-value
		Yes	No		Yes	No	
Lumbar	Normal	16 (5.3)	113 (37.7)	0.53	13 (4.3)	116 (38.7)	0.80
	Osteopenia	17 (5.7)	104 (34.7)		11 (3.7)	110 (36.7)	
	Osteoporosis	5 (1.7)	45 (15)		4 (1.3)	46 (15.3)	
Femoral	Normal	5 (1.7)	79 (26.3)	0.65	8 (2.7)	76 (25.3)	0.67
	Osteopenia	19 (6.3)	96 (32)		12 (4)	103 (34.3)	
	Osteoporosis	14 (4.7)	78 (29)		8 (2.6)	93 (31)	

Data are presented as number (%).

The association between thyroid dysfunctions and BMD in the femoral and lumbar areas is presented in Table 3. Among 28 patients with thyroid dysfunctions, in their lumbar area 11 subjects (3.7%) were osteopenic and 4 subjects (1.3%) were osteoporotic and in the femoral area, 12 subjects (4%) were osteopenic and 8 subjects (2.6%) were osteoporotic which was not statistically significant in both areas (P-value >0.05).

The association between BMI, DM, and thyroid dysfunctions with BMD in linear regression model has been presented in Table 4. In the unadjusted and adjusted model only the association of BMI with BMD in the lumbar area (B-unadjusted: 0.013 and B-adjusted: 0.02) and in the femoral area (B-unadjusted: 0.004 and B-adjusted: 0.006) were statistically significant.

**Table 4:** **The association of BMI, DM, and thyroid dysfunctions with BMD in linear regression in lumbar and femoral areas**

		B^1^unadjusted	SE	P	B adjusted^2^	SE	P-value
Lumbar area	BMI (kg/m²)	0.013	0.002	<0.01	0.02	0.002	<0.01
	Thyroid dysfunctions (Y/N)	0.014	0.03	0.69	0.013	0.03	0.65
	DM (Y/N)	.038	0.03	0.26	0.05	0.03	0.11
Femoral area	BMI (kg/m²)	0.004	0.002	0.04	0.006	0.002	<0.01
	Thyroid dysfunctions (Y/N)	-0.028	0.03	0.36	-.023	0.02	0.39
	DM (Y/N)	-0.025	0.03	0.38	0.001	0.02	0.91

^1^Regression coefficient.

^2^Adjusted for age, sex.

## Discussion

In the present study, in lumbar and femoral areas, the mean BMI of osteoporotic subjects is lower than normal and osteopenic subjects and also underweight subject are more prone to osteoporosis. These findings are consistent with Amiri et al. study which showed that BMI is associated with osteoporosis and osteopenia [22].

In the present study, no association was found between DM and BMD, which is consistent with Pajouhi et al. [23]. In contrast, Albright & Reifenstein study [24] and Mima et al. [25] showed that DM is associated with osteoporosis. The Moghimi et al. study showed that diabetic women had lower T-Score and were more prone to osteoporotic in comparison with healthy women [26]. Also Roe et al showed that vertebral bone density in insulin-dependent diabetic children is lower than that in healthy children [27]. Several studies in children and adults population support this finding [28, 29]. In contrast, other studies showed that BMD in type II DM subjects is higher or equal to normal population [30–32]. In another study carried out in Greece, it was shown that both types of DM exacerbate osteoporosis [33].

The results of BMD in type I and II DM subjects is different or even contradictory [8–11]; so that in some studies there was no difference between BMD in subjects with type II DM and healthy subjects, while in a number of other studies, lower BMD and still in others higher BMD has been observed [8, 10]. The latter was more often observed in women, which has been attributed to obesity and the increased androgeneity due to hyper Insulin- dependency [7]. The findings of our study regarding the independence of thyroid dysfunctions from BMD are in contrast with findings reported by Folis [20]. He showed that hyperthyroidism causes bone erosion, declaring that this change in the bone metabolism is accompanied by degradation of collagen tissue and reduction of the bony density. The role of thyroid hormones in bone metabolism is not clarified, but the suggested mechanism involves the impact of T3 on the osteoclasts causing bone erosion [20]. Likewise, other studies showed that thyroid dysfunctions would increase the risk of osteoporosis [33, 34]. The low number of subjects with DM and thyroid dysfunctions in our study population is the main limitation of this study. The cross sectional design is another limitation of this study.

## Conclusion

The results of current study show that underweight subjects are more prone to osteoporosis, but no association was found between DM and thyroid dysfunctions with osteoporosis.
